# Venous Thromboembolism in Burn Patients: A 5-Year Retrospective Study

**DOI:** 10.3390/medicina60020258

**Published:** 2024-02-02

**Authors:** Eliza-Maria Bordeanu-Diaconescu, Andreea Grosu-Bularda, Adrian Frunza, Sabina Grama, Mihaela-Cristina Andrei, Tiberiu Paul Neagu, Cristian-Sorin Hariga, Ioan Lascar

**Affiliations:** 1 Burn Centre, Emergency Clinical Hospital of Bucharest, 014461 Bucharest, Romania; eliza.diaconescu@umfcd.ro (E.-M.B.-D.); andreea.grosu-bularda@umfcd.ro (A.G.-B.);; 2 Department of Plastic Surgery and Reconstructive Microsurgery, ”Carol Davila” University of Medicine and Pharmacy Bucharest, 010825 Bucharest, Romaniaioan.lascar@umfcd.ro (I.L.)

**Keywords:** burns, venous thromboembolism, complications

## Abstract

*Background and Objectives:* Burn patients manifest all components of Virchow’s triad, amplifying the concern for venous thromboembolism (VTE). Routine prophylaxis for VTE remains a subject of debate, with the central concern being the occurrence of associated adverse events. *Materials and Methods:* We conducted a five-year retrospective study on burn patients admitted to our burn center. Demographic data, comorbidities, burn lesions characteristics, surgical interventions, anticoagulant medication, the need for transfusions, the presence of a central venous catheter, length of stay, complications, and mortality were recorded. *Results:* Of the overall number of patients (494), 2.63% (13 patients) developed venous thromboembolic complications documented through paraclinical investigations. In 70% of cases, thrombosis occurred in a limb with central venous catether (CVC). Every patient with VTE had a Caprini score above 8, with a mean score of 12 points in our study group. *Conclusions:* Considering each patient’s particularities and burn injury characteristics, individualized approaches may be necessary to optimize thromboprophylaxis effectiveness. We suggest routinely using the Caprini Risk Assessment Model in burn patients. We recommend the administration of pharmacologic thromboprophylaxis in all patients and careful monitoring of patients with Caprini scores above 8, due to the increased risk of VTE. Additionally, ongoing research in this field may provide insights into new strategies for managing thrombotic risk in burn patients.

## 1. Introduction

Venous thromboembolism (VTE), including both deep venous thrombosis (DVT) and pulmonary embolism (PE), is a severe medical condition linked to significant morbidity and mortality. Deep vein thrombosis (DVT) involves the development of blood clots within the deep veins, typically occurring in the lower extremities. The acute phase of DVT poses a notable risk, as the clot may travel to the pulmonary circulation, resulting in pulmonary embolism (PE) and potentially impairing cardiopulmonary function. Beyond the immediate risk of pulmonary embolism, venous thromboembolism can lead to long-term complications, including chronic thromboembolic pulmonary hypertension and post-thrombotic syndrome [1,2,3].

Although venous thromboembolism (VTE) may occur without an evident major cause, commonly referred to as “unprovoked” VTE, the majority of VTE cases are associated with one or more identifiable risk factors. These factors can either be linked to the patient’s characteristics, often of a permanent nature, or to acute clinical conditions, typically of a transient nature, which can contribute to or facilitate the onset of VTE [4,5].

Preventive measures and early detection are crucial in managing DVT. Patients at risk, such as those with a history of venous thrombosis, prolonged immobility, or certain medical conditions, may be prescribed anticoagulant medications. Additionally, lifestyle modifications such as maintaining physical activity and using compression stockings, can help reduce the risk of DVT [6,7].

Venous thromboembolic complications in burn patients present a significant clinical concern, largely influenced by the unique pathophysiological changes that occur following a burn injury.

Burns patients exhibit all factors of Virchow’s triad:

- Stasis due to immobilization following grafting procedures and prolonged hospitalization, undergoing major general surgery (the risk rises with increasing procedure duration > 45 min), and association with other major trauma;
- Endothelial damage due to persistent inflammation, multiple operative procedures, and placement of intravenous lines;
- Hypercoagulability: burns induce a hypercoagulable state, caused by circulating clotting factors and platelets [8,9,10,11].

Despite this high theoretical risk, there is considerable variability within different studies reporting VTE in burn patients, with VTE prevalence ranging from 0.2% to 25% [12]. The American Burn Association (ABA) statistics emphasize the heightened risk for venous thromboembolism in burn patients. The reported incidence of DVT was 0.48%, that of PE was 0.18%, and that of VTE was 0.61%. When the extent of the burn exceeds 10% of the total burn surface area (TBSA), the risk for DVT increases to 0.92%, for PE to 0.38%, and for VTE to 1.2%, according to ABA investigation. Among burn patients admitted to the intensive care unit (ICU), the incidence of venous thromboembolism was 1.2%, and this rate was significantly higher compared to burn patients who did not require admission to the ICU. These data underscore the importance of recognizing and addressing the risk of DVT in burn patients, particularly in those with larger burn areas [13].

However, it is crucial to note that the actual incidence of VTE in burn patients may be underreported, as many instances of deep venous thrombosis (DVT) and pulmonary embolism (PE) can be clinically silent and challenging to detect [14].

Duplex ultrasonography is widely accepted as the primary diagnostic procedure for the evaluation of suspected deep vein thrombosis and is also commonly used for follow-up assessments to monitor the response to anticoagulant therapy or to identify the progression of DVT. CT pulmonary angiography (CTPA) has become the primary imaging modality for diagnosing pulmonary embolism due to its high sensitivity and specificity. It provides detailed and cross-sectional images of the pulmonary vasculature. Although a CT scan may not be very accurate in detecting small peripheral emboli, it is capable of revealing various structures in the thorax, providing a comprehensive view of the chest area, and can reveal conditions other than thromboembolism, assisting in identifying alternate diagnoses [15].

## 2. Methods

We retrospectively reviewed the hospital records of the burn patients admitted to the Burn Centre of Bucharest Emergency Clinical Hospital over 5 years (October 2018 to September 2023) and identified patients with deep venous thrombosis and/or pulmonary embolism. The data collected included age, gender, comorbidities, injury history including mechanism and associated trauma, percentage of total body surface area (TBSA) and burn degrees, distribution of lesions, length of hospital stay, operative history, anticoagulant medication, the need for transfusion (red blood cell transfusions, platelet transfusions, and plasma transfusions), and the placement of central venous access. Patients with incomplete medical records or missing documentation of thromboembolic complications were excluded from the study. The obtained data were recorded in a Microsoft Excel database.

The Caprini thrombosis risk assessment scale was used to evaluate the estimated risk of thrombosis in these patients [8]. We employed a simplified formula due to the inability to assess patients for personal or family history of positive blood tests indicating an increased risk of blood clotting (factor V Leiden, prothrombin gene mutation, protein C and S deficiency, lupus anticoagulant, antiphospholipid antibody, Beta2 glycoprotein, antithrombin III—testing is not available in our burn center).

The Abbreviated Burn Severity Index (ABSI) score was also calculated [16].

The body mass index (BMI) was computed, and individuals with a BMI equal to or exceeding 30 kg/m^2^ were categorized as obese.

For extensive burns (>15% TBSA), intravenous fluid resuscitation was initiated at admission according to the Parkland formula proposed by Charles R. Baxter and was promptly adjusted to adequate perfusion endpoints to prevent under or over-resuscitation. According to this formula, the total fluid volume to be administered during the initial 24 h was calculated as 4 mL of Lactated Ringer’s solution multiplied by the patient’s weight (in kilograms) multiplied by the percentage of TBSA affected by the burn injury. Half of the calculated fluid volume was infused during the first eight hours, starting from the time of the initial burn. Fluid resuscitation was adapted to maintain a urine output of over 0.5 mL/kg/h.

Routine burn wound care was performed daily, and burn wounds were dressed according to our standardized unit protocols.

The decision regarding the CVC insertion site was individualized based on the circumstances of each case, including the extent and location of the burn injuries, the patient’s overall condition, and the specific needs for medical treatment. A careful aseptic technique was used during the insertion and maintenance of the CVCs.

Upon admission to the burn center, pharmacological and mechanical thromboprophylaxis was initiated. Anticoagulant medication was routinely used and consisted of enoxaparin 40 mg subcutaneously once a day. In individuals who were bedridden or had limited mobility, VTE prophylaxis included changing body positions in bed regularly, performed by caregivers, passive limb function exercises, and kinesiotherapy. Whenever feasible, patients were encouraged to participate in active limb function exercises and commence early mobilization, transitioning from supine position to sitting, standing, or ambulation. In patients who underwent surgical excision and skin grafting, mobility was restricted postoperatively for 3 to 5 days.

Burn wound infections, cases of pneumonia, bloodstream infections, and urinary tract infections were documented, and the American Burn Association Sepsis Criteria were used to define septic patients [17].

The diagnosis of DVT was made in our burn center using duplex ultrasound at the patient’s bedside, with a focus on clinically symptomatic patients presenting with increased limb edema or pain. All patients with DVT underwent screening for PE. CT pulmonary angiography was used for the diagnosis of PE, providing an assessment of the location and degree of clot burden. In every patient diagnosed with DVT, weekly ultrasound examinations were conducted to monitor the size and evolution of the thrombus under treatment.

All the procedures of this study respect the ethical standards in the Helsinki Declaration of 1975, as revised in 2008. Written consent was obtained from the patients for publication of the accompanying images.

## 3. Results

We identified 494 patients with burn injuries who were admitted to our burn center from October 2018 to September 2023. Out of the 494 patients, 13 patients (2.63%) developed venous thromboembolic complications that were documented through paraclinical investigations (either ultrasound or computerized tomography scan) (Table 1). Additionally, two patients were excluded from the study because, although the echocardiography raised the suspicion of pulmonary thromboembolism, the patients died before the computed tomography scans could be performed.

All 13 patients received central venous catheters (CVC) upon admission to facilitate access to fluid resuscitation, nutritional support, and the administration of medications (Table 1).

Out of the 13 patients, 10 patients had symptomatic DVT (2.02% of the total patients included in the study), characterized by unilateral swelling of the limb, with tenderness or pain. We registered one case of deep vein thrombosis at the level of the upper limb; in the remaining nine cases, the lower limb was affected. Duplex ultrasound was used to diagnose DVT and the entire venous system of the affected extremity was assessed for compressibility, augmentation, and intraluminal echoes. A duplex ultrasound was also performed on the contralateral limb, but we did not detect any bilateral lower extremity thrombosis. At the moment of diagnosis of venous thromboembolism, the mean level of D-dimers was 1627.66 ng/mL. Serial ultrasound examinations were conducted weekly to monitor the size and evolution of the thrombus under treatment.

One patient with DVT was also diagnosed with pulmonary embolism. In another patient, PE was suspected based on clinical findings and echocardiography, but unfortunately, the patient died before a CT scan examination.

**Figure 1:**
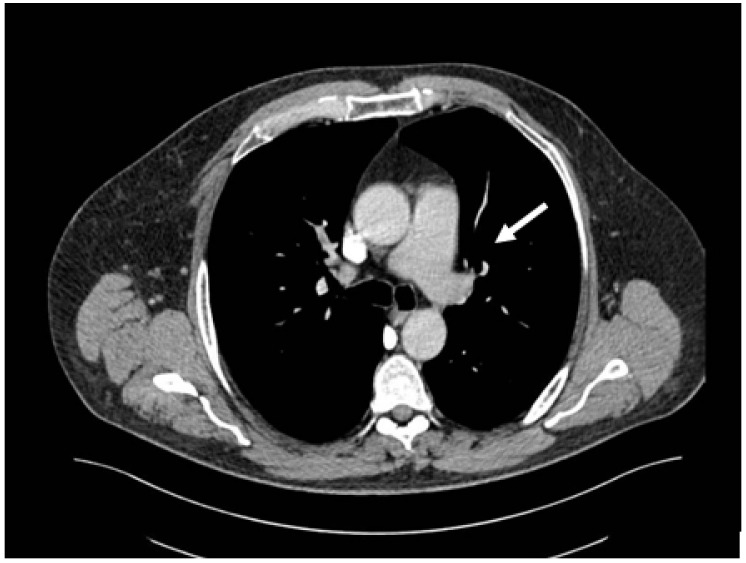
CT pulmonary angiography—pulmonary embolism, thrombus on the left main pulmonary artery (white arrow).

Out of the 13 patients, 3 patients had PE without evidence of DVT (0.60% of the total patients included in the study) (Figure 1).

The mean age of the population with VTE was 52.38 years (SD = 13.71) (Table 2).

Assessment or personal or family history of positive blood tests indicating an increased risk of blood clotting was not available in our burn center, so a simplified Caprini thrombosis risk assessment scale was used. The mean Caprini score was 12 points. In our study, 6 out of the 13 patients (46.15%) with thromboembolic complications had a BMI ≥ 30 kg/m^2^. (Table 1).

Among the 13 patients, 3 patients had a history of malignancy: a patient with stage 4 pancreatic cancer and hepatic metastases was diagnosed with PE, a second patient with stage 4 hepatocellular carcinoma and pulmonary metastases was diagnosed with iliofemoral DVT, and a third patient with a history of cervical neoplasm, without evidence of metastatic disease at the moment, was diagnosed with iliofemoral–popliteal DVT.

**Figure 2:**
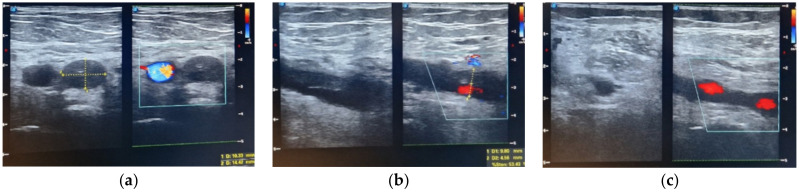
Duplex ultrasonography (**a**) iliac vein thrombosis, (**b**) femoral vein thrombosis, (**c**) popliteal vein thrombosis in resolution (day 25 after thrombosis).

**Figure 3:**
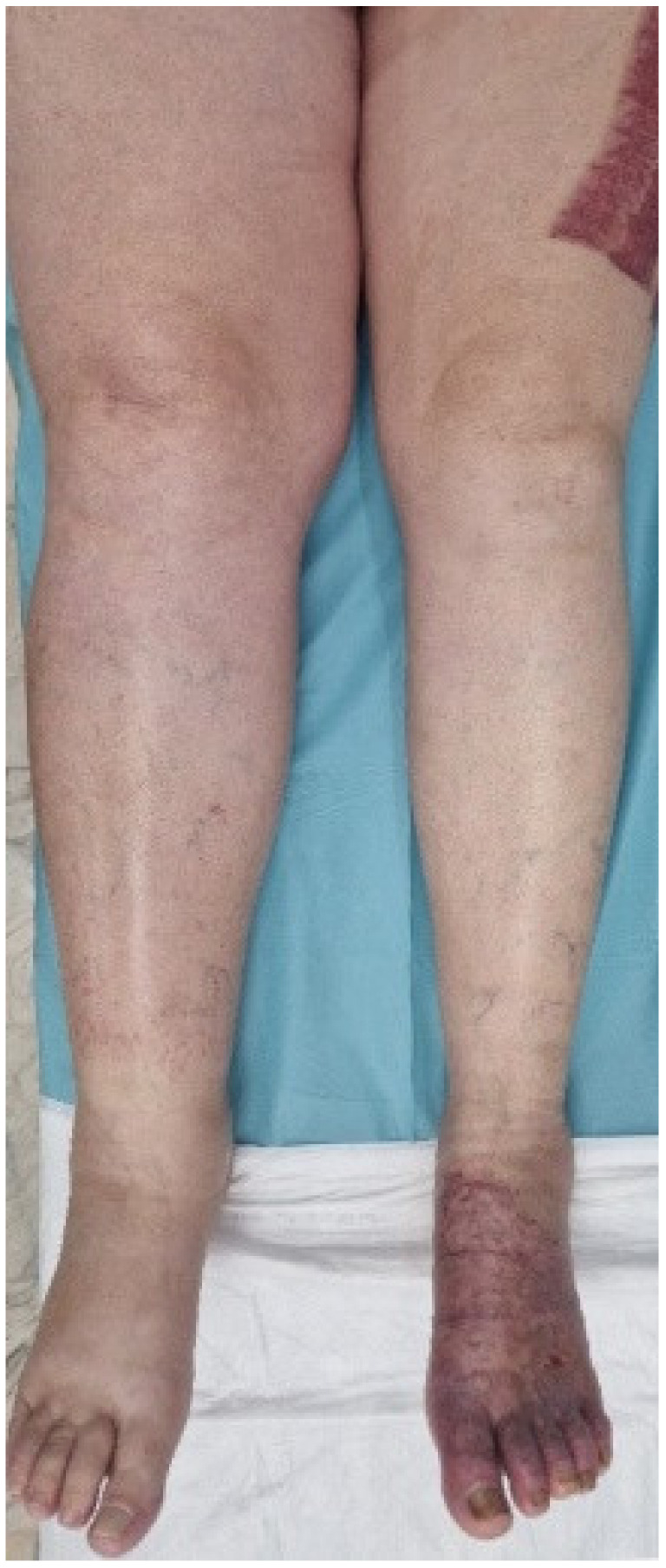
A clinically symptomatic patient presenting with increased limb edema.

The average ABSI score was 8 points. The average TBSA was 30.38%, with TBSA between 1% and 75% TBSA. The patient with 1% TBSA had a femoral venous catheter placed on the limb affected by deep vein thrombosis, and the Caprini score was 14. The patient was obese, suffered from diabetes and hypertension, and had a history of cervical cancer 3 years before the burn injury, treated by total hysterectomy and bilateral adnexectomy, external beam radiation therapy, and brachytherapy (Figure 2 and Figure 3). The patient with 4% TBSA had DVT on the donor limb for split-thickness skin grafts. The Caprini score was 14, the patient was obese and suffered from VHB/VHC co-infection, liver cirrhosis, hepatocarcinoma stage IV (lung and bone metastases), and followed a chemotherapy regimen.

The mean interval between the burn injury and the diagnosis of VTE was 27.92 days, with one case of PE diagnosed on the CT scan in the emergency room in the first 24 h after the burn trauma.

The average length of hospitalization was 44.83 days, and in six patients (46.15%), the occurrence of the venous thromboembolic complication increased the number of days of hospitalization.

In our study, six patients (46.15%) were intubated and mechanically ventilated before the venous thromboembolic complication appeared, with an average of 11.08 days of mechanical ventilation (between 3 days and 50 days).

Inhalation injury, diagnosed by bronchoscopy, was present in three patients (23.08%) with VTE.

In seven cases of patients with DVT (70%), thrombosis occurred in a limb with CVC, and CVC was immediately removed from that site.

In five cases of patients with DVT, thrombosis occurred on a burned extremity (50%).

Only one patient had concurrent traumatic injuries. This patient had a humerus fracture and underwent orthopedic surgery and immobilization. On day 34 after the burn injury, the patient was diagnosed with pulmonary embolism, with a thrombus on the right upper lobe pulmonary artery.

The mean number of surgeries was 2.61, including escharotomies or fasciotomies and excision and grafting, depending on the specific characteristics of the burn injury and the patient’s overall condition. Surgical procedures were performed under regional or general anesthesia, and each operation lasted for more than one hour. Mobility was restricted postoperatively for 3 to 5 days, depending on the surgical site, in the 11 patients who underwent burn excision and skin grafting.

Out of the 13 patients, 5 patients had a history of mental illnesses, with low compliance to treatment and difficult cooperation during passive limb function exercises and kinesiotherapy.

We identified three patients with DVT who also had sepsis at the moment of diagnosis.

The overall mortality was 30.77% (four patients) in our study group, with pulmonary embolism being the suspected cause of death in two cases, and sepsis in the other two cases.

All survivors were discharged after evidence of thrombus decrease on ultrasonography for DVT and thrombus resorption on CT scan for PE and received long-term oral anticoagulation with apixaban.

## 4. Discussion

The occurrence and risk factors related to the development of venous thromboembolism in burn patients remain a subject of debate. Thromboembolic events might be underdiagnosed in burn patients due to the challenges in clinical assessment, the masking of the main clinical signs of DVT caused by the burn, such as edema and tenderness in the limbs, and the focus on managing the burn itself [14]. In clinical studies that involve screening asymptomatic patients using duplex ultrasound, a significant proportion of DVT cases in burn patients seemed clinically occult, with studies reporting an incidence between 6 and 23%. However, the clinical importance of asymptomatic DVT remains uncertain [13].

Data from the literature on general trauma and medical patients recommend that in cases of suspected deep vein thrombosis (DVT), diagnostic testing should begin with an evaluation of the clinical pretest probability. If the clinical pretest probability is low and the D-dimer test yields a negative result (typically <500 ng/mL), DVT can be excluded. In cases where the clinical pretest probability is moderate or high, ultrasound imaging should be conducted without measuring the D-dimer [18]. On the contrary, an analysis of burn patients by Ahuba Rb et al. [19] concluded that D-dimer test results are not a useful screening tool for DVT in patients with burn injuries because of extremely low specificity and a low positive predictive value. At the moment of diagnosis of venous thromboembolism, the mean level of D-dimers in our study group was 1627.66 ng/mL.

In our burn center, ultrasound screening for deep vein thrombosis is not routinely performed and diagnostic tests are obtained when there is a clinical suspicion. In this analysis, it was found that 2.63% of patients experienced VTE complications. Among these, 2.02% were diagnosed with deep vein thrombosis, while 0.60% were diagnosed with pulmonary embolism. A study by Panucci et al. [20], which included the data of 22,618 adult patients from the American Burn Association’s National Burn Repository, reported an overall incidence of VTE of 0.97%. In similar studies, the rate of DVT ranges from 0.25% [21] to 5.92% of patients [22], and PE is reported in 0.05% of patients [21], depending on the injury-specific risk factors and whether or not patients who experienced prolonged inactivity or needed mechanical ventilation support for an extended time were routinely screened for DVT with duplex ultrasound.

Multiple studies have shown an increased risk of VTE in patients with malignancy, with prevalence as high as 20% [23]. The intricate relationship between cancer and venous thromboembolism (VTE) is underpinned by a complex interplay of mechanisms, leading to a hypercoagulative state. This prothrombotic state includes a massive release of inflammatory cytokines triggered by cancer cells, the expression of hemostatic proteins on the surface of tumor cells, the activation of the coagulation cascade by procoagulant substances released by tumor cells, and the activation of platelets and other components of the clotting system [3,24,25,26,27]. Studies have reported a 4- to 7-fold higher risk of VTE in patients with cancer [28]. The American Society of Hematology 2021 guidelines advocate for thromboprophylaxis in hospitalized medical patients with cancer, using low molecular weight heparins (LMWH) or fondaparinux for surgical patients with cancer [29]. We routinely administer thromboprophylaxis using LMWH in our burn patients (enoxaparin 40 mg subcutaneously once a day). A particular finding is that 3 of the 13 patients with VTE had a history of malignancy, and they developed thromboembolic complications despite the fact they received thromboprophylaxis.

In our study, 70% of the patients with deep vein thrombosis had DVT that appeared on the limb where the central venous catheter was inserted (six femoral vein CVCs and one subclavian vein CVC). Previous studies found a notably elevated thrombosis rate in femoral catheterization compared to subclavian catheterization, and the odds ratio for femoral vein-associated thrombosis was reported to be 14.42 [30]. Given that central venous catheterization is frequently necessary for burn patients, studies have suggested that opting for an upper extremity site is preferable, as the risk of embolization from the upper extremity ranges from 2% to 9%, significantly lower than the 29% risk associated with the lower extremities [31,32].

Only 50% of DVT cases appeared on a burned extremity. Given the preliminary findings of this small study, we believe that the distribution of burn wounds is less important than other factors of Virchow’s triad in the occurrence of DVT in hospitalized burn patients.

Burn patients with sepsis commonly experience coagulation disorders. As a result, they face an elevated risk of thrombotic complications, ranging from subclinical coagulopathy to fulminant disseminated intravascular coagulation (DIC) and the occurrence of venous thromboembolism [33,34]. Septic patients commonly display a prothrombotic state characterized by extrinsic pathway activation, cytokine-induced coagulation amplification, anticoagulant pathway suppression, and fibrinolysis impairment. Monocytes and macrophages, when activated, release cytokines and chemokines, subsequently activating neutrophils, platelets, and endothelial cells. This activation leads to the release of tissue factor into the circulation, thereby activating the extrinsic coagulation pathway. Neutrophils, in addition to their role in the immune response, express tissue factor and release chemical mediators, significantly contributing to the activation of the coagulation. In the early stages of sepsis, three key anticoagulant pathways play a crucial role in regulating coagulation during sepsis: antithrombin, protein C, and tissue factor pathway inhibitor, yet these mechanisms are deranged as sepsis progresses. The dysregulation of anticoagulant pathways, coupled with continued proinflammatory responses, results in a shift toward a hypercoagulable state [35,36]. In our study, three patients with deep vein thrombosis also had sepsis at the time of diagnosis of the thromboembolic complications.

Recent research has revealed that receiving RBC transfusions, regardless of the type, is linked to heightened chances of experiencing pulmonary embolism (PE) and deep vein thrombosis (DVT), due to increased hematocrit levels after RBC transfusion. The risk is higher when RBC transfusion is combined with a transfusion of fresh frozen plasma [37]. Six patients with VTE in our study received RBC transfusions with a mean number of 6.5 units of RBC, and among them, four patients also received fresh frozen plasma, with a mean number of 3.25 units. Previous research on burn patients demonstrated that the transfusion of more than 4 units of red blood cells was associated with VTE [38].

The majority of our patients faced an increased risk of thromboembolism due to immobility or limited range of motion following skin grafting procedures. Additionally, these patients experienced reduced mobility due to pain-related restrictions in their range of motion. Previous studies also identified immobility as a major risk factor for venous thromboembolic disease in burn patients [39].

Previous research postulated that mechanical ventilation in burn patients is associated with approximately two-fold odds of developing DVT [40]. We observed that almost half of the VTE cases in our burn center (46.15%) appeared in patients who were mechanically ventilated, rendering passive mobilization difficult. Although, the initiation of early mobilization for patients in the intensive care unit is deemed safe and beneficial, incorporating early mobility into standard clinical care practices poses challenges [41,42]. Patient-related barriers to early mobilization we encountered were hemodynamic instability, vascular access devices, the presence of tubes and drains, the administration of sedative drugs, decreased level of consciousness, and non-compliant behavior of patients due to psychiatric disorders. We also identified structural barriers: a limited number of staff and limited equipment. Expanding our physiotherapy would be beneficial in implementing early rehabilitation programs [43].

Obesity is commonly acknowledged as a risk factor for venous thromboembolism (VTE). Despite obesity being recognized as a moderate risk factor for venous thromboembolism (VTE), its impact can be influenced by interactions with other risk factors in both the development and recurrence of VTE, such as genetic factors, hormone replacement therapy, systemic inflammation, and insulin resistance [44,45]. While certain studies in the literature have identified obese burn patients as having a substantial risk for VTE [46], this association is not consistently observed across all studies [38,39,47]. In our study, 6 out of the 13 patients (46.15%) with thromboembolic complications presented a BMI ≥ 30 kg/m^2^.

Experts have not reached a consensus regarding the use of routine prophylactic subcutaneous heparin or LMWH administration. Some authors consider that routine prophylaxis can lead to adverse events, such as bleeding and heparin-induced thrombocytopenia, and should be reserved for obese patients, patients with a history of DVT or PE, lower extremity burns, burn wound infection, and associated trauma or orthopedic injury [40]. Other authors support the prophylaxis of burn patients, given the prevalence of VTE in their studies and the evidence supporting the use of VTE prophylaxis in surgical settings, emphasizing its efficacy, safety, and cost-effectiveness [21,38]. A survey investigating 21 burn units in the German-speaking part of Europe found that the risk of heparin-induced thrombocytopenia (HIT) and DVT were higher in burn centers using unfractionated heparin intravenously (HIT rate of 2.7% and DVT rate of 3.8%) than in burn centers using LMWH subcutaneously (HIT rate of 0.2% and DVT rate of 0.9%) [48]. In our burn center, we administer prophylactic low-molecular-weight heparin (enoxaparin 40 mg subcutaneously once a day) in all burn victims and have only noticed very few significant side effects.

As part of the initial management strategy, the American Society of Hematology (ASH) 2020 guidelines recommend, for patients with pulmonary embolism (PE) and hemodynamic compromise, the use of thrombolytic therapy followed by anticoagulation as opposed to anticoagulation alone. In the majority of patients with proximal deep vein thrombosis (DVT), the recommendation is for anticoagulation therapy alone, without the addition of thrombolytic therapy. Top of FormAfterward, it is advised to maintain primary treatment with anticoagulant therapy for a total duration of 3 to 6 months for VTE caused by transient risk factors. Secondary prevention with antithrombotic therapy in patients with DVT and/or PE provoked by a chronic risk factor should be followed indefinitely, and direct oral anticoagulants (DOACs) are preferred over vitamin K antagonists (VKAs). Patients should undergo regular reevaluation to assess the benefits and risks of ongoing anticoagulant therapy [49]. All the survivors in our study were prescribed DOACs (rivaroxaban or apixaban) for 3 to 6 months at discharge, with further follow-ups conducted periodically to assess whether the continuation of anticoagulation therapy is necessary.

The GARFIELD-VTE study showed elevated rates of mortality, recurrent venous thromboembolism (VTE), and bleeding within the initial year following a VTE diagnosis, especially within the first month, highlighting the crucial significance of prompt and effective treatment for newly diagnosed patients with VTE [50].

Through its extensive validation in over 250,000 patients across more than 100 clinical trials worldwide, the Caprini Risk Assessment Model has proven to be a valuable tool in guiding healthcare professionals in assessing and managing VTE risk in diverse patient populations. The Caprini score assigns specific point values to each risk factor, and the cumulative score helps categorize individuals into different risk levels. A higher score indicates an elevated risk of VTE [33,51,52]. In our study, all the VTE patients had Caprini scores above 8, with a mean score of 12 points. The results are in concordance with previous studies, which recorded an increased risk for VTE in burn patients with Caprini scores higher than 8. Regularly assessing the Caprini score in burn patients proves beneficial for prospectively stratifying the risk of venous thromboembolism (VTE) following admission and determining appropriate prophylactic measures [53].

## 5. Conclusions

In conclusion, individuals with burn injuries are exposed to an increased susceptibility to venous thromboembolism, influenced by various contributing elements (immobility, hypercoagulability, endothelial dysfunction, patient-specific factors, such as age, comorbidities, and genetic predispositions). Considering each patient’s unique characteristics and burn injury, individualized approaches may be necessary to optimize thromboprophylaxis effectiveness. We advise central venous catheter insertion via the jugular vein, the subclavian vein, or the veins of the upper limb, taking into account the lower risk for catheter-related venous thrombosis. We suggest routinely using the Caprini Risk Assessment Model, commonly called the Caprini Score, in burn patients. We recommend the administration of pharmacologic thromboprophylaxis in all patients and careful monitoring of patients with Caprini scores above 8, due to the increased risk of VTE. Additionally, ongoing research in this field may provide insights into new strategies for managing thrombotic risk in burn patients.

## Figures and Tables

**Table 1:** Characteristics of study patients with venous thromboembolic complications.

Patient	Age	Sex	BMI ≥ 30	Day of VTE after Burn Injury	ABSI Score	Caprini Score	%TBSA	Depth	Inhalation Injury	DVT	PE	CVC Placement
1	56	f	no	40	11	10	50	IIa–IIb–III	yes	right iliac thrombosis	no	left femoral
2	48	m	yes	3	8	9	40	IIa–IIb–III	no	no TVP	yes	right femoral
3	63	f	yes	24	7	14	1	IIB–III	no	right iliofemoral and popliteal thrombosis	no	right femoral
4	46	m	yes	6	7	8	40	IIa–IIb	no	right iliac thrombosis	no	right subclavian
5	39	f	no	46	9	10	45	IIa–IIb–III	no	right subclavian thrombosis	no	right femoral
6	65	f	yes	13	7	14	4	IIa–IIb–III	no	right iliofemoral thrombosis	no	left femoral
7	40	m	no	54	8	16	32	III	yes	left femoral and popliteal thrombosis	no	right femoral
8	74	f	no	34	7	16	15	IIa–IIb	no	no TVP	yes	right jugular
9	80	m	no	1	13	13	75	IIb–III	no	no TVP	yes	left femoral
10	50	f	yes	39	9	12	35	III	no	left femoral thrombosis	suspected on echocardiography	left femoral
11	46	m	no	11	5	10	8	III	no	right iliac thrombosis	yes	right femoral
12	34	m	yes	27	4	12	10	III	no	right femoral and popliteal thrombosis	no	right femoral
13	40	f	no	65	9	12	40	IIb–III	yes	right iliac and femoral thrombosis	no	right femoral

BMI: body mass index, VTE: venous thromboembolism, ABSI: Abbreviated Burn Severity Index, TBSA: total body surface area, DVT: deep vein thrombosis, PE: pulmonary embolism, CVC: central venous catheter.

**Table 2:** Demographics and clinical characteristics of study patients with venous thromboembolic complications.

Variables	Classification	Cases	Proportion (%)
Sex	Female	7	53.85
	Male	6	46.15
Age	<40	2	15.38
	40–60	7	53.85
	60–80	4	30.77
Mechanism of injury	Flame	10	76.92
	Contact burns	1	7.69
	Scalds	2	15.38
Burn setting	Accident	9	69.23
	Self-harm	4	30.77
%TBSA	<20	5	38.46
	20–40	5	38.46
	41–60	2	15.38
	61–80	1	7.69
3rd degree burns	No	1	7.69
	Yes	12	92.31
Burn site	Head and neck	10	76.92
	Torso	9	69.23
	Upper limbs	10	76.92
	Lower limbs	7	53.85
Smoker	Yes	6	46.15
	No	7	53.85
Alcoholism	Yes	4	30.77
	No	9	69.23
CVC insertion	Femoral vein	11	84.61
	Jugular vein	1	7.69
	Subclavian vein	1	7.69

## Data Availability

Dataset available on request from the authors.
